# Cost minimisation analyses of birth care in low-risk women in Norway: a comparison between planned home birth and birth in a standard obstetric unit

**DOI:** 10.1186/s12913-024-11631-7

**Published:** 2024-09-30

**Authors:** Pål Joranger, Anette Schaumburg Huitfeldt, Stine Bernitz, Ellen Blix

**Affiliations:** 1 Department of Nursing and Health Promotion, Faculty of Health Sciences, Oslo Metropolitan University, Pilestredet 32, Oslo, 0130 Norway; 2 Oslo University Hospital, Forskningsveien 2b, Oslo, 0373 Norway; 3 Department of Obstetrics and Gynaecology, Østfold Hospital Trust, Kalnesveien 300, Grålum, Sarpsborg 1714 Norway

**Keywords:** Cost minimisation analysis, Maternity care, Childbirth, Home birth, Hospital birth, Being on call

## Abstract

**Background:**

Evidence exists that planned home births for low-risk women in settings in which they have access to hospital transfer if needed are safe. The costs of planned home births, compared to low-risk births in obstetric units, are not clear. The aim of this study was to compare costs associated with hospital births versus home births under different home birth organizations.

**Methods:**

We performed a cost minimisation analysis (CMA) based on decision-analytic modelling while assuming that health outcomes were not affected by place of birth. Estimations of resource use were mainly based on three existing Norwegian datasets: (1) women with planned home births (*n* = 354), (2) women with planned home births (*n* = 482) of which 63 were transferred to a hospital, and (3) women with planned births in a hospital (*n* = 1550).

**Results:**

Planned home birth costs 45.9% (credibility interval [CrI] 39.1–54.2) of a low-risk birth at a hospital. For planned home birth, the birth was the costliest activity (32.1%). The costs for planned home birth were estimated to be €1872 (CrI 1694–2071) and included hospitalisations for some. Costs for only those with actual home birth was €1353 (CrI 1244–1469). Costs of a birth, including possible birth-related complications, in low-risk women in a hospital was €4077 (CrI 3575–4615).

When including the costs of being on call for one woman at a time, a planned home birth costs €5,531 (CrI 5,171–5,906), which is 135.7% (CrI 117.7–156.8) of low-risk births at a hospital. When organizing midwives in the on call teams for multiple women at a time, a planned home birth costs € 2,842 (CrI 2,647–3,053), which is 69.7% (CrI 60.3–80.9) of a low-risk birth in a hospital.

**Conclusions:**

Home birth can be cost-effective if the midwives who facilitate home births are organised into larger groups, or they work for hospitals that also facilitate home births. A model in which midwives work separately or in pairs to assist with a home birth and are on call for one birth at a time may not be cost-effective.

**Supplementary Information:**

The online version contains supplementary material available at 10.1186/s12913-024-11631-7.

## Introduction

Evidence exists that planned home births for low-risk women in settings in which they have access to transfer to a hospital if needed are safe and associated with a reduction in interventions, such as caesarean sections and assisted vaginal deliveries [1–3]. ‘Low risk’ is usually defined as healthy women with a singleton foetus in a cephalic position, spontaneous onset of labour, gestational age between 37 and 42 weeks, and no complications or a previous caesarean section. In some settings women are define as high-risk during the late term, namely starting from gestational week 41.

The costs of planned home births and whether they cost less than low-risk women giving birth in obstetric units are not clear. A systematic review that investigated the costs of births in alternative settings [4] included four studies that compared costs between home birth and hospital settings and found that a home birth cost less than a hospital birth. The authors highlighted that differences in health systems, methods of analysis, and resources included in the costs are present [4].

In economic analyses that assess the costs of home births, the definition of the home birth group will have a major impact on the costs. The home birth group may consist of only women who gave birth at home. Alternatively, this group may consist of both women who gave birth at home and women who intended to give birth at home but were transferred to hospital after 37 weeks gestational age, which is the timepoint from when the home birth provider(s) in Norway are on call. In addition, this group can include women who planned for a home birth but changed their minds or were advised to give birth in a hospital before 37 weeks of gestational age.

Anderson and Anderson [5] compared women who actually had an uncomplicated home birth with women who had an uncomplicated hospital birth. They found that the costs of a home birth were 32% those of a hospital birth. On the other hand, Hitzert et al. [6] based the selection of groups on women’s *planned* place of birth. Of those who planned for a home birth, 45.6% of nulli-parous women and 84.6% of the multi-parous women succeeded in their plan and gave birth at home [6]. Hitzert et al. found that in a Dutch setting, the birth costs for those who initially planned for home birth was 90% of the adjusted mean costs for those who initially planned for hospital birth. Similar inclusion criteria for the two groups were used in Hendrix et al. [7] in which the women were asked about their *preferred* place of delivery, either at home or in a hospital. Of those who preferred a home birth, 64.3% gave birth in hospital, while 76.8% of those who initially preferred to give birth in hospital gave birth in a hospital. They found that the birth costs in the Netherlands for those who initially selected home birth were 94% of the costs for those who initially preferred hospital births.

Whether the analysis includes the costs of being on call or not also seems to be important. An Italian study by Cicero et al. [8] found that home births were associated with 50% less costs when compared with hospital births if the public health service provided the home birth. No costs associated with being on call were included. Additionally, these authors performed analyses of home births in a private health care setting in which the costs of being on call were included and found that the costs of home births were 59% higher than those for hospital births. It is not clear whether the costs of being on call was a major reason for the large differences between publicly and privately offered home births.

Approximately 55,000 births annually in Norway have been reported of which 100–150 (2–2.5/1000) are home births [9]. Health services are financed through taxes, and all intrapartum care is public and free of charge, except for home births provided by independent midwives. National guidelines for planned home births were previously established [10]. The health authorities do not organise home births, so women must find a midwife willing to assist. The Norwegian Labour and Welfare Organisation pays the midwife for assisting a birth but not for the costs for being on call or for the transport to the woman’s home; those costs are covered by the woman.

To the best of our knowledge, no previous studies have conducted an economic evaluation that compared the costs of planned home birth with planned birth in an obstetric unit in Norway or another Nordic country. Furthermore, previous studies have scarce descriptions on what tasks midwives do before, during and after home births. If health authorities or others want to organise home births, it is important that the costs of the service are known.

The aim of our study was to perform a cost minimisation analysis (CMA) to compare the costs of births planned at home versus those associated with birth in an obstetric unit in low-risk women.

We addressed the challenges of selecting the groups by comparing the costs of the location in which they actually gave birth (home versus hospital) and by selecting the women for home and hospital births late in their decision-making process (when a woman may sign a contract with a midwife for a home birth), so relatively few change their planned place of birth.

Furthermore, we analysed the costs of being on call and how different ways of organising home birth assistance affects these costs. We also describe the costs associated with the various activities by the midwives before, during, and after the home birth.

## Methods

### Setting for maternity care in Norway

In Norway, maternity care is free of charge, health services are financed through taxes, and healthcare staff receive fixed salaries. Except for home births, intrapartum care is provided in public institutions. Intrapartum care is organised at three levels: (1) highly specialised units with a neonatal intensive care unit that provide advanced paediatric, obstetric, and anaesthetic services; (2) units in smaller hospitals with obstetric and anaesthetic services; and (3) free-standing and alongside midwifery-led units providing care for low-risk women only. Healthy women with low risk of complications can give birth at all three levels. The mean duration of a hospital stay after a vaginal birth is 2.4 days [11]. The health authorities do not organise home births; thus, a woman planning for a home birth must make an agreement with independent midwives. The labour and welfare administration will cover the expenses for intrapartum care but not for the midwife being on call or for the transportation to and from the woman’s home. One, sometimes two, midwives would be on call for every single woman.

### Design and perspective

The economic evaluation performed was a cost minimisation analysis (CMA), and we used a model approach to compare costs [12]. Costs representing equipment and time spent on interventions (such as caesarean sections, assisted vaginal deliveries) and complications (such as postpartum haemorrhage, perineal tears) were included in the analyses. We assumed that health effects, such as health-related quality of life and mortality, are not affected by place of birth [1–3]. Organisation of data and the cost analysis were done by using decision-analytic modelling [13, 14].

In the cost analysis, we focused on the cost differences between planned home births and births at a standard obstetric unit. The care associated with a birth is generally assumed to be similar for both birth procedures, so these costs were not included in the analysis. However, a standard premise for home birth is that the midwife and the pregnant woman become relatively well-known/familiar before birth. This process may imply antenatal home visits, other meetings, or telephone calls. Data concerning this kind of extra activity were collected from the midwives.

### The alternatives

We compared the costs of giving birth in a hospital with giving birth at home. We used two alternatives for hospital births:

#### A1 low-risk birth in hospital

According to our data, a low-risk birth is assessed as low-risk upon the onset of labour. Some of them will develop risk factors or complications during labour and may receive treatment, such as assisted vaginal delivery or caesarean section. These women stay in the hospital for at least 24 h.

#### A2 vaginal birth in hospital

All births are vaginal births with no major complications, and the mothers stay in the hospital for at least 24 h. This procedure means that both mother and baby are treated in agreement with the least expensive procedures in terms of score according to diagnostic-related group (DRG codes 373 and 391, respectively). The A2 alternative includes, for example, the use of forceps and vacuum during birth. On the other hand, when a greater amount of bleeding (> 1500 ml) or sphincter ruptures occur, the birth is considered complicated and thus is reclassified as DRG 372 and alternative A1.

Alternatives A1 (described above) and B1 (described below) are the main alternatives that are compared as they are based directly on the three data sets used in this study. Using the other alternatives (A2, B2, B3, and B4) to compare hospital births with home births is done to analyse the effect of change assumptions and the way of organising home birth. For home births, we chose two other alternatives:

#### B1. Planned home birth

Women who planned for a home birth and made an agreement with one or more midwives to assist at approximately 37 weeks of gestational age. This alternative includes both women who gave birth at home and women transferred to hospital before, during, or after birth.

#### B2. Home birth

Women who planned for a home birth, who gave birth at home, and without transfer after the birth.

We also analysed models for home birth that include the costs of an on call midwife. The cost estimations of being on call are heavily dependent on which assumption we made about how the midwives are organised and how being on call is valued, and how the pay for being on call varies, which can range from from one to three to one to seven hours. Different ways midwives assisting home births organise being on call have been used and range from one midwife being on call for one woman to a group of midwives sharing on being on call for a group of women. To address this difference, we used a micro-simulation model to simulate the on call costs for different models and combinations. Of these simulated combinations, we chose to focus on these two alternatives and compared them with the costs of a hospital delivery:

#### B3. Planned home birth + on call for single women

**Fig. 1:**
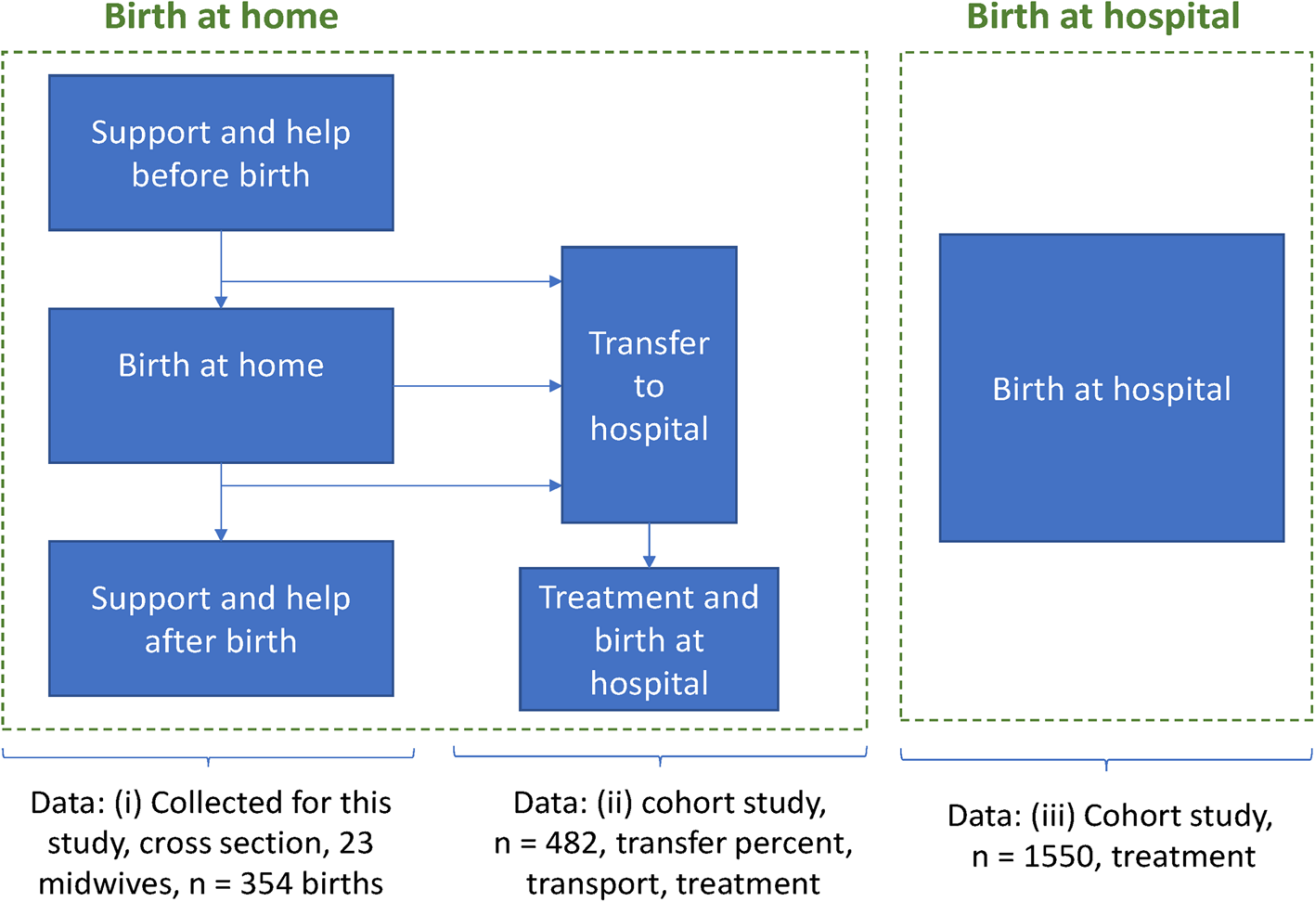
The main data sources for the resource use data

Women who plan to give birth at home and make an agreement with one or more midwives to assist. Such an agreement is inclusive of the costs for these midwives to be *on call* for one woman at a time. This alternative is based on our data from sources (i) and (ii) and is shown in Fig. 1.

#### B4. Planned home birth + on call for groups

Women who plan to give birth at home and make an agreement with a *group of midwives* to assist. This agreement includes the costs for the midwives to be on call for a group of women.

For alternative B4, we used experiences from the Caseload Midwifery model tested at Karolinska University Hospital [15] to make the assumptions about being on call. In this model, 16 midwives were organised on four teams and handled both deliveries in hospital and at home. This model established that each midwife, on average, should take care of 40 pregnant woman per year. In Norway, we have an average of 45.2 working weeks per year, so if we apply this model in a Norwegian context, each midwife is then assumed to have the main responsibility for 0.89 (40 deliveries per year/45.2 weeks per year) deliveries per week on call. Furthermore, we assumed that two midwives needed to be on call so that the midwife with the main responsibility for a birth can be backed up by another midwife if needed.

### The sample, data, and variables for the resource-use data

The study was conducted in Norway, and we used three datasets: (i) *Data collected from midwives attending home births (‘Home birth data’)*. Midwives attending home births in Norway are organised in a loose network with an e-mail list they can use to contact each other for questions, discussing experiences, and organising meetings. We contacted the members of the network in June 2017, which consisted of 28 active midwives at that time. Midwives who had attended at least four home births from January 1st, 2010, to December 31st, 2017, were asked to participate. Twenty-three of the 28 midwives fulfilled the inclusion criteria, and all of them consented to participate in the study. They were asked to estimate the time spent on several tasks for the last 4–40 home births they had attended during the study period: (1) preparing equipment for birth, (2) home visits and other meetings before the birth, (3) time spent during labour and birth, (4) home visits and other meetings after the birth, and (5) time spent on transportation to and from the woman’s home or on accompanying her to the hospital. Assessments of time used were based on information in the patients’ files. We asked how many midwives were involved with each woman and the time each of them spent with her. The midwives recorded the woman’s age (< 30, 30–35, > 35 years), parity (primi- or multi-parous), whether she had previously given birth at home (yes/no), education level (under or equal to high school, or above), the distance from the woman’s home to the nearest hospital (0–5 km, 6–10 km, 11–20 km, 21–30 km and > 30 km), and the distance from the midwife’s home to the woman’s home (0–10 km, 11–30 km, > 30 km). We asked the midwives about their experiences as home birth midwives (years), the position percentage as home birth midwife (5–100% for which full time is 37.5 h per week), size of position in a hospital or municipality (0–100%), how many planned home births they had assisted from 2010 to 2017, including those transferred to hospital during or after labour and birth, and how many women that had booked a home birth with them but for which the woman either changed her mind about place of birth or was transferred to hospital before the onset of her labour. The questionnaires we used in this study can be found in Additional file no. 1. These questionnaires were developed for this study and have not been previously published elsewhere.

(ii) *Data for those planned for home birth but transferred to hospital (‘Transfer data’)*: Anonymised data from a previous study were used in the analyses [9]. The study reported outcomes and transfers to and treatments in hospitals in a Nordic cohort of women who planned for home births. We extracted the Norwegian data from a study of 482 women who planned for a home birth between 2008 and 2012 and where 63 (13.1%) were transferred to hospital.

(iii) *Data for planned birth at hospitals (*‘*Hospital birth data*’*)*: Anonymised data from a previous study containing a cohort of 1,550 women who planned for a hospital birth from 2008 to 2012 [9]. The women were at low risk of complications, which is defined as the spontaneous onset of labour, with a singleton foetus in a cephalic position, no chronic medical diseases before pregnancy (epilepsy, heart diseases, hypertension, kidney diseases, diabetes), no complications during pregnancy (vaginal bleeding after 28 weeks of gestation, pregnancy-induced hypertensive disorders, gestational diabetes), no previous caesarean section or foetal death before labour onset, and gestational age between 37 and 42 weeks.

Data collected for samples (ii) and (iii) included maternal age (< 30, 30–35, > 35 years), marital status (married/cohabitant or single), smoking (no/yes), body mass index (< 25, 25–29.9, *≥* 30), parity (primi- or multi-parous), previous caesarean section (yes/no), birthweight (< 1500, 1500–4499, ≥ 4500 g), mode of birth (spontaneous vaginal, assisted vaginal, or caesarean section), and estimated maternal blood loss (< 500, 500–1499, ≥ 1500 ml). For the home birth group (ii), whether the woman or baby (or both) were transferred to hospital, what vehicle was used for transfer, and transfer duration (≤ 30, 31–60, > 60 min) were recorded.

In data set iii, no data for the women’s level of education were available. As a proxy in Table 1, we used the education level in 2016 among all women in Norway at the same ages (weighted for the three levels of age).

**Table 1:** Characteristics of the three main study samples

	**Home birth data **(i) ***N*** *= 354 (%)*	**Planned home birth, with 63 transferred to hospital **(ii) ***N*** *= 482 (%)*	**Hospital birth data **(iii) ***N*** *= 1550 (%)*
**Age in years**			
< 29	102 (28.8)	142 (29.6)	816 (52.7)
30–34	140 (39.6)	187 (40.0)	468 (30.2)
> 35	112 (31.6)	151 (31.5)	365 (17.1)
**Education**			
Low	92 (26.0)	127 (26.6)	(50.3)^a^
High (Univ.)	262 (74.0)	350 (73.4)	(49.7)^a^
**Km from midwife to woman**			
0–10 km	112 (31.6)		
11–30 km	130 (36.7)		
31 + km	112 (31.6)		
**Km from woman to hospital**			
0–5 km	85 (24.0)		
6–10 km	61 (17.2)		
11–20 km	85 (24.0)		
21–30 km	63 (17.8)		
31+ km	59 (16.7)		
**Minutes per transport to hospital**			
≤ 30 min		50 (82.0)	
31–60 min		10 (16.4)	
> 60 min		1 (1.6)	
**Previous birth**			
No	79 (22.3)	90 (18.7)	733 (47.3)
Yes	275 (77.7)	391 (81.3)	817 (52.7)
**Previously caesarean section**			
No		471 (97.7)	1550 (100.0)
Yes		11 (2.3)	0 (0.0)
**Smoking**			
Yes		16 (3.3)	157 (11.9)
No		464 (96.7)	1157 (88.1)
**BMI**			
< 25		311 (72.5)	480 (71.1)
25–29.9		93 (21.7)	144 (21.3)
≥ 30		25 (5.8)	51 (7.6)

^a^Education level in 2016 among all women in Norway at the same ages (weighted); *BMI* body mass index

### Unit costs

The unit costs are reported in Table 4. For the estimation of home birth cost, the costs of the midwife’s working time is important. For estimating the alternative cost for midwives normal working hours (planned visits), we used a Norwegian midwife’s average salary inclusive of income taxes (€ 47,551 per year) and added social costs and employer’s tax (30.51% of gross salary).

For midwives who work at random times of day and days of the week, we added a mark-up to the hourly wage. This markup is relevant as midwives can be randomly called out for a birth or ‘false alarms’ at any time of the day or day of the week, including night hours and weekends. In the healthcare system, staff are compensated with extra pay for working these hours. Transport costs were estimated based on national tariffs per km used for transport in private care and marked prices for the use of taxis per km. For car, boat, and helicopter ambulances, we used estimates from Oftedahl [16] and Statistics Norway.

For resources used in hospitals, the estimation of unit cost was based on the diagnostic-related group (DRG) and the DRG-weight for the relevant interventions.

### Statistical analysis

For many of the cost components of the simulation, we used an estimate for the average probability of using a certain resource (such as, the average probability of having a second meeting/consultation with the midwives before birth at home), and for those who use it, we estimated the amount used (such as the average time use for this meeting among those who had this meeting). We used dummy variables to estimate the unadjusted probability and its standard error. Unadjusted mean value and its standard error and confidence interval (CI) were estimated for the resource use among those that used the resource (such as minutes used per meeting for the second meeting before birth at home). The type of distributions with associated values are reported in Additional file 2.

We also performed a subgroup analysis based on alternative B1 to determine how the composition of the groups of women giving birth could affect the use of resources. The methods used and results are presented in Additional file 2.

The analyses were performed in SPSS 28 and Stata 17.

### The model and assumption for estimating on call costs

To estimate the costs of being on call, we partly based the simulation on empirical data and partly on assumptions about how society chooses to organise midwives. We assumed that the due date is day 282 and that on call starts three weeks before the due date and can last at most until two weeks after the due date. Thus, on call starts on day 261 and ends on day 296. Based on the probability in Norway of giving birth on the various days during this period, we estimated the average number of days on call to be 19.57 (data set ii). Furthermore, the compensation for healthcare professionals being on call varies across Norway. Therefore, we used a simulation model to estimate costs in different situations.

Three conditions are important to the costs of being on call:

1. The compensation for being on call. In Norway, this compensation is often expressed in terms of the number of hours a person must be on call to get paid for one of these hours, typically 1:3, 1:5, or 1:7.
2. Number of births in each defined on call period (how many days on call per birth).
3. The number of midwives who are on call for a given birth.

Regarding condition 1, the alternative cost we included was welfare loss (defined as reduced individual quality of life) for the midwives to be on call. Possible salary loss for the midwives or loss in production for society by being on call were excluded. The costs of midwives rushing out to assist women in labour was estimated elsewhere in the main model. We used the compensation in the Norwegian health service for being on call as a proxy for this welfare loss. The compensation is stated as the number of hours a midwife must be on call to get paid for one of these hours. In Norway, this number of hours varies normally from 7 to 3 but for which we estimated 5 h as the most appropriate for our group of midwives. Due to uncertainty about the compensation level, we estimated the on call costs and based the cost on different compensation levels.

Furthermore, we used the midwife’s net income (gross income minus 27%) as compensation because that is the part of the compensation from the society that corresponds to their welfare loss. Because they are on call every hour all week, we added 24.7% to their salaries to also include compensation for overtime pay and public holiday supplement.

Based on data from Blix et al. [9], we found that an average on call period lasts for 19.57 days per birth per midwife.

### Time horizon, currency, and conversion

The time horizon was one year because all costs related to the different alternative evaluated were generated for one year. We therefore did not need to adjust future costs to their present value, and consequently, did not need to discount the costs. All costs were estimated in 2019 Euros, and we used purchasing power parity (PPP) to convert from Norwegian kroner (NOK) to Euro. One NOK was estimated to be € 0.07188.

### Uncertainty and sensitivity analysis

To determine the uncertainty in the results, we performed both deterministic and probabilistic sensitivity analyses (DSA and PSA, respectively). The simulation model was constructed using Excel (Office version 2306), and @Risk 7.6 was used for performing the PSAs.

In the DSA, we showed the variations in costs depending on variations in the assumptions related to being on call by varying the level of compensation for being on call and the number of women giving birth per week per midwife on call (see Table 7).

Total parameter uncertainty in the input variables was analysed using the PSA for which all uncertainties in the relevant parameters were considered simultaneously. The distributions used in the PSA for the resources used were mainly based on analysis of patient-level data. PSA simulations for probabilistic scenarios were also performed to handle structural uncertainty by designing and analysing the costs of alternative ways of organising midwives who facilitate home births. Results are presented with a 95% credibility interval (CrI), which shows the 2.5th and 97.5th percentile of the outcome distribution from the PSAs.

In the PSA, we used the gamma distribution for costs and resource parameters and beta distributions for probability parameters. The distributions of the parameters were based on the confidence interval (CI) and standard error (SE) estimated for our patient-level data and the use of Briggs et al. [13] to estimate the alpha and beta values we applied in the distributions. See also Additional file 3 on the defined distributions and other information on the choice of distributions for the PSA.

### Ethics

The project was approved by the Norwegian Centre for Research Data (Registration No. 447238/25.03.2020).

## Results

The age, education level, and parity were about the same for the two home birth groups (‘Home birth data’ and ‘Transfer to and treatment in hospitals data’ as shown in Table 1). We had no data on education for the women who planned for hospital birth but used the 2016 education level among all women in Norway at the same ages as a proxy. Based on these data, we found a difference between the home birth and hospital birth groups regarding age, education level, and the proportion of previous births. The women in the home birth group were older, had higher levels of education, and more of them had previously given birth. The effects of these differences were analysed separately in subgroup analysis.

The hospital birth group had undergone no previous caesarean sections (was an inclusion criterion for a low-risk birth/woman) compared to 2.3% of the home-birth group (Table 1). The home-birth group had fewer smokers (3.3% versus 11.9%), and the two groups had approximately the same body mass index (BMI) distribution.

### Input data to the simulation model

In Table 2, input data of the simulation model for planned home birth are presented. We see that 96.9% of the women had a contract meeting, and among those who had such a meeting, the meeting lasted on average for 2.60 h. On average, for the birth, midwife no. 1 (the main midwife) was at a woman’s home for 7.13 h, and in 52.5% of the births, the no. 2 midwife (the second midwife if present) was also there and contributed for 5.77 h.

**Table 2:** Resource used for planned home birth (the base case). The source was the home birth data (see Fig. 1, data i)

Component	Probability of the event to occur	Unit per meeting/ delivery	The mean for the events that occurred
**Birth at home**			
Time used for transport to the woman		Hours/visit (both ways)	1.146
Km from the woman in labour to the hospital		Km (one way)	21.28
Contract meeting (CM)	0.969	Hours	2.601
* Meetings between CM and birth*			
1^st^ meeting	0.424	Hours	1.828
2^nd^ meeting	0.294	Hours	1.673
3^rd^ meeting	0.226	Hours	1.592
4^th^ meeting	0.127	Hours	1.694
5^th^ meeting	0.045	Hours	2.119
6^th^ meeting	0.017	Hours	2.128
False alarm:*			
The trip	0.325	Hours	3.922
Arrange the equipment	0.325	Hours	1.196
* The birth:*			
Midwife 1	1	Hours	7.131
Arrange the equipment	1	Hours	1.196
Midwife 2	0.525	Hours	5.774
Arrange the equipment	0.525	Hours	1.196
* Meetings after birth:*			
1^st^ meeting	0.972	Hours	1.613
2^nd^ meeting	0.667	Hours	1.523
3^rd^ meeting	0.300	Hours	1.878
4^th^ meeting	0.085	Hours	3.079
5^th^ meeting	0.031	Hours	2.944
6^th^ meeting	0.006	Hours	2.944
Check by a paediatrician at home	0.8644	Hours	1.0
Of those checked by a paediatrician: Check at home	0.2695		
Of those checked by a paediatrician: Check at hospital	0.7305		
Organising the paediatrician’s visit	0.8644	Hours	0.458
Outpatient consultation** (time used by midwife)	0.0226	Hours	1.6875
Other time used by the midwife per birth***	0.576	Hours	2.752

* The midwife travelled to the woman but returned as the birth had not started. ** Maternal or neonatal complications requiring that the midwife to organize a visit to the hospital outpatient clinic

***: Telephone consultations before and after the birth

Among women who initiated home birth, 8.71% were transferred to a hospital during birth, and 2.49% were transferred after birth (Table 3). When a woman was hospitalised after birth, she was in the hospital for two days (based on expert opinions). Of those who were transferred to hospital, 35.38% used ambulances (car or boat) and 3.08% used ambulance helicopters. Of the women transferred before birth, 21.43% were estimated to have had a caesarean section. For all the planned home births, this transfer constitutes 1.87% (0.2143 × 0.0871 × 100) compared to 5.87% for the planned hospital births (Table 3). The hourly costs for a general practitioner (GP) as shown in Table 4 is based on the Norwegian Medicines Agency’s unit costs [17], and the costs of outpatient consultation (DRG915O) were retrieved from the Norwegian results-based financing system [18].

**Table 3:** Data for planned hospital birth and for transfer and hospitalisation of women and children with a planned home birth

Component	Probability of the event to occur ^c^	Data sources^d^
**Transfer to hospital during and after planned home birth**		
Urgent hospitalisation before birth, mother	0.0871	ii data
Newborns transferred alone or with mother	0.1099	ii data
*For those transported to hospital*:		ii data
By private care	0.6000	ii data
By taxi	0.0154	ii data
By ambulance	0.3538	ii data
By ambulance helicopter	0.0308	ii data
*Hospital*,* for the transferred mothers before birth (conditional probabilities*^*e*^*)*:		ii data
Caesarean section w/cc^a^ (DRG 370)	0.0000	ii data
Caesarean section no/cc^b^ (DRG 371) (unconditional probability^f^: 0.01867)	0.2143	ii data
Vaginal delivery w/cc (DRG 372) (unconditional probability: 0.0127)	0.146	ii data
Vaginal delivery no/cc (DRG 373) (unconditional probability: 0.0556)	0.639	ii data
*Hospital*,* for the transferred newborn* *(conditional probabilities)*:		ii data
Healthy newborn, no/cc (DRG 391) (unconditional probability: 0.1011)	0.9800	ii data
Newborn, birth weight 1500–2499 g or other immaturity, without multiple prob. (DRG 388B) (unc. prob.:0.00220)	0.0200	ii data
Urgent hospitalisation of mother after birth	0.0249	ii data
**Planned for birth at hospital**		
*Hospitalised mother*:		
Caesarean section w/cc (DRG 370)	0.0000	iii data
Caesarean section no/cc (DRG 371)	0.0587	iii data
Vaginal delivery w/cc (DRG 372)	0.0394	iii data
Vaginal delivery no/cc (DRG 373)	0.902	iii data
*Hospitalised newborn*:		iii data
Healthy newborn, no/cc (DRG 391)	0.9961	iii data
Newborn, birth weight 1500–2499 g or other immaturity, without multiple prob. (DRG 388B)	0.0039	iii data

*DRG* diagnosis related group

^a^: w/cc = with complications or comorbidities

^b^: no/cc = No complications or comorbidities

^c^: To find the ‘Probability of the event to occur’, we calculated the proportion of women who received a certain treatment or shown a particular event from our data

^d^: i, ii, and iii referred to the dataset shown in Fig. 1

^e^: conditional probabilities indicate that the probabilities apply given that the woman has been transferred to give birth in hospital

^f^: unconditional probability indicates the probability based on all women that panned for home birth

**Table 4:** Unit prices used in the cost analysis

**Component**	**Unit**	**Costs **	**Data source**
**Home birth costs**:			
Midwife time cost	€/hour	36.61	Estimated
Midwife time cost in case of emergency	€/hour	45.66	Estimated from OUS
GP	€/hour	60.95	(17)
Outpatient consultation	€ per consultation	86.69	DRG915O (18)
Transport to the woman, car costs	€/visit (both ways)	18.65	Estimated from Home birth data and national tariff
Time cost for transport to the woman	€/visit	14.86	Estimated.
Equipment	€/birth	25.23	Experts
Medication	€/birth	15.93	Experts
* Transport to hospital when emergency:*			
Privat care	€/transport	6.18	Tariff
Taxi	€/transport	47.92	Marked prices
Ambulance	€/transport	528.89	Ref. 15
Helicopter	€/transport	4,147.69	Ref. 15
**Hospital costs, mother**:			
Caesarean section w/cc	€	6,352.05	DRG370 (19)
Caesarean section no/cc	€	4,544.98	DRG371 (19)
Vaginal delivery w/cc	€	3,049.24	DRG370 (19)
Vaginal delivery no/cc	€	2,015.71	DRG371 (19)
**Hospital costs, newborn:**			
Healthy newborn, no/cc	€	1,842.38	DRG391 (19)
Newborn, birth weight 1500–2499 g or other immaturity, without multiple prob.	€	9,414.14	DRG388B (19)

*w/cc* with complications or comorbidities, *n/cc* No complications or comorbidities

Experts: Two midwives with long experience with home births. *GP* general practitioner, *OUS* Oslo University Hospital

### Cost components of home birth

We estimated the costs of the different activities related to an average birth at home as shown in Table 5. For the planned birth at home (B1), the birth at home (travel costs and time use for the midwives traveling and involvement during the birth at home) was found to be the costliest activity (32.1%) followed by the costs of treating hospitalised women and children (25.8%). The costs of being transported to the hospital constitutes 2.3% of the total cost, and this value includes the use of one’s own car, taxi, ambulance, and ambulance helicopter. When estimating the costs of each actual transfer (the transport cost), this actual transfer costs was € 319 (95% CrI 163–606). The average cost for the activities for women who deliver at home was relatively similar to the average cost for those who planned to deliver at home. Medications and equipment represent 3.1% of the total costs of birth at home (see Additional file 4 for more on medications and equipment costs).

**Table 5:** Costs (in Euros) of activities related to an average home birth

Activities	Planned home birth (B1)		Home birth (B2)	
	Costs (€)	95% CrI	Costs (€)	95% CrI
Contract meeting (CM)	126.8	115.7–138.6	126.4	115.2–138.4
Meetings between CM and birth	112.6	100.6–125.5	105.2	93.0–118.3
‘False alarms’	70.5	55.8–87.2	58.0	44.6–73.6
Birth, midwife 1	415.8	373.4–461.0	436.8	388.9–488.0
Birth, midwife 2	185.8	157.5–216.5	182.4	155.1–212.3
Meetings after birth	202.3	185.3–219.4	249.7	186.2–220.9
Check by a paediatrician	111,8	101.2–124.9	127.6	116.2–139.4
Outpatient consultation	3.8	1.6–6.9	3.9	1.54–7.27
Other time used for the midwife	58.0	48.0–69.3	51.3	41.9–61.9
Medication and equipment	58.1	42.6–79.8	58.1	42.6–79.8
Treatment in hospital, woman	264.6	185.4–361.5	0.0	-
Treatment in hospital, newborn	219.1	152.1–305.6	0.0	-
Transport to hospital	43.0	20.8–84.4	0.0	-

### Comparing the costs of different places of birth

We estimated the total incremental costs and cost differences between a planned birth in a hospital and a birth at home (Table 6). The costs of an average vaginal hospital birth without complications (A2) was € 3,858 (CrI 3.332–4.423), and the costs of an average birth in low-risk women with possible complications (A1) who were in the hospital was € 4,077 (CrI 3.575–4.615). This finding indicates that the latter costs 5.7% more than an uncomplicated vaginal birth. Costs for both the woman and child were included. An average planned home birth (B1) was estimated to be € 1,872 (CrI 1.694–2.071), and the costs of an average actual home birth (B2) were € 1,353 (CrI 1.244–1.469). The costs of the planned home birth (B2) were higher because some of the women or children were hospitalised before, during, or after delivery (see also Tables 3 and 4).

**Table 6:** Total incremental costs and cost differences between a planned birth in the hospital versus at home

**Total costs for the hospital and home birth alternatives (CrI)**					
Low-risk birth in hospital (A1)	Vaginal birth in hospital (A2)	Planned home birth (B1)	Home birth (B2)	Planned home birth + on call (B3)	Planned home birth + on call for groups (B4)
**4**,**077** (3.575 to 4.615)	**3**,**858** (3.332 to 4.423)	**1**,**872** (1.694 to 2.071)	**1**,**353** (1.244 to 1.469)	**5**,**531** (5.171 to 5.906)	**2**,**842** (2.647 to 3.053)
**Differences in costs between home and hospital births: Hospital birth costs – home birth costs**					
*The hospital birth alternatives that the home birth alternatives are compared with*:		Planned home birth (B1)	Home birth (B2)	Planned home birth + on call (B3)	Planned home birth + on call for groups (B4)
Hospital births, all low-risk women (A1)		**2**,**204** (1,669 to 2,774)	**2**,**724** (2,209 to 3,274)	**-1**,**454**^**a**^ (-803 to -2,077)	**1**,**235** (637 to 1,811)
Hospital births, low-risk women with uncomplicated vaginal births (A2)		**1**,**986** (1,428 to 2,580)	**2**,**505** (1,967 to 3,080)	**-1**,**673**^**a**^ (-1002 to -2,313)	**1**,**016** (693 to 1,617)
**Home birth costs as a percent of hospital birth costs: (Home birth costs/hospital birth costs) x 100**					
*The hospital birth alternatives that the home birth alternatives are compared with*:		Planned home birth (B1)	Home birth (B2)	Planned home birth + on call (B3)	Planned home birth + on call for groups (B4)
Hospital birth, all low-risk women (A1)		**45.9%** (39.1 to 54.2)	**33.2%** (28.6 to 38.8)	**135.7%** (117.7 to 156.8)	**69.7%** (60.3 to 80.9)
Hospital birth, low-risk women with uncomplicated vaginal births (A2)		**48.5%** (40.9 to 57.9)	**35.1%** (29.9 to 41.5)	**143.4%** (123.0 to 168.0)	**73.7%** (63.1 to 86.6)

B1: Home birth, Low-risk women, all births. Including women transferred and received hospital treatment

B2: Home birth, Low-risk women, only those who actually gave birth at home

B3: As B1 + the costs for the midwives to be on call for one woman at a time (the Norwegian settings)

B4: As B1 + the costs for some midwives to be on call for more than one woman at a time. The midwives were organised in larger groups to assist during home birth (based on a model from Karolinska University Hospital, Swedish)

^a^: Minus means that home births cost more than hospital birth for this comparison

If we compare all low-risk hospital births (A1) with planned home births (including possible complications for birth at hospital and at home, B1), the hospital births cost € 2,204 (CrI 1, 669–2,774) more than the home births (Table 6). This finding indicates that the planned home birth (B1) costs 45.9% (39.1–54.2%) of a low-risk birth at hospital (A1). Even if we compare planned home births with possible complications (B1) and vaginal births without complications in a hospital (A2), home births were found to be € 1,986 (CrI 1,428–2,580) less costly and constituted 48.5% (CrI 40.9–57.9%) of hospital births.

An uncomplicated (without complications or comorbidities, w/cc, in Tables 3 and 4) vaginal birth in a hospital (A2) was found to cost € 2,505 (CrI 1,967–3,080) more than an uncomplicated home birth with no transfer to hospital (B2). This home birth (B2) represents 35.1% (CrI 29.9–41.5%) of the costs of hospital-based vaginal birth without complications (A2).

The results for planned home birth + on call (B3) and planned home birth + on call for groups (B4) in Table 6 did not include the costs of being on call. This finding is in line with most the other comparable studies. Under some circumstances, being on call can be excluded from the analyses, typically when large maternity wards at hospitals without extra on call costs can also facilitate home births and send out midwives who are on duty when a need for assistance at a home birth arises.

### Including being on call in the comparisons of costs

Under other circumstances/organizations than those mentioned above, the costs for the midwives to be on call can be of importance for the total costs of home birth. Three conditions are important for the costs of being on call: (1) Compensation for being on call, (2) how many days on call per birth, and (3) the number of midwives who are on call for a given birth. See the methods section in which these assumptions and the choice of values used in the analysis are addressed.

In Table 7, the costs of different combinations of 1 and 2 above are presented. The costs of being on call varies considerably with changes in the assumptions. For example, the combinations of 0.358 births per midwife per week and compensation of five hours to work to get paid for one of these hours, the costs per birth for one midwife would be € 2,397 (CrI 2,223–2,575). This number (0.358) represents the number of births per week if a midwife is on call for 19.57 days on average while waiting for one birth. This cost was 28.0% higher than all other costs for a planned birth at home. If two midwives are on call per birth, the costs would be doubled to € 4,794, and if, on average, 1.525 midwives are on call as assumed in the alternative ‘Planned home birth + on call’ (B3), the on call costs would be € 3,655.

**Table 7:** Costs per midwife on call related to compensation and the number of births per midwife per week

		**Number of births per midwife per week**			
		**0.358**	**0.885**	**1.000**	**2.000**
**On call compensation: The number of hours to work to get paid for one of these hours**	**3**	€ 3,995	€ 1,616	€ 1,430	€ 715
	**5**	€ 2,397	€ 970	€ 858	€ 429
	**7**	€ 1,712	€ 693	€ 613	€ 306

The number of 0.885 births per midwife per week illustrates the on call costs if using the model from Karolinska University Hospital in Sweden (see the methods section) in our Norwegian setting. Based on this model, the on call costs per birth was € 970 (CrI 900–1,042). In Table 6, this cost is included in the alternative ‘Planned home birth + on call for groups’ (B4).

The level of compensation, the number of midwives on call, and the number of births that the midwives contribute to per week vary widely between practices. To illustrate the total costs of home births, including on call costs, when compared with the costs of hospital births, we selected the alternatives shown in Table 6 (see the discussion of these in the methodology section).

We see that “Planned home birth + on call” (B3) category has a total cost of € 5,531 (CrI 5,171–5,906), which is € 1,454 (CrI 803–2,077) more than the costs for low-risk birth at hospital (A1). On the other hand, by organising the midwives as teams on which they handle more births per week, we see that the cost for “Planned home birth + on call for groups” (B4) is € 1,235 (CrI 637–1,811) less than low-risk birth at hospital (A1). This finding means a 30.3% lower costs per home birth than for low-risk births in hospital.

### Distribution of costs among the women who give birth at home

**Fig. 2:**
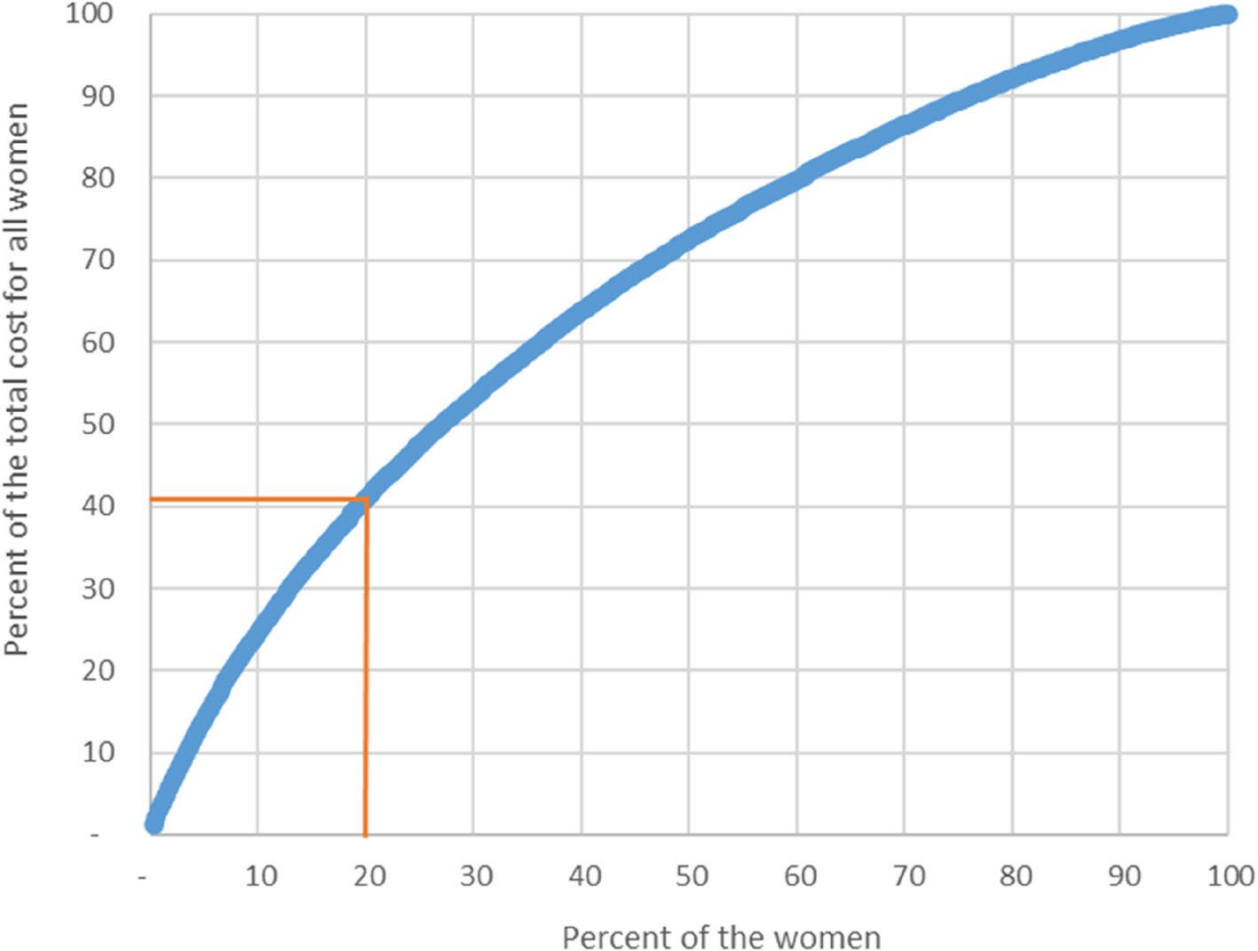
Distribution of the midwife time cost (exclusively travel time costs and costs of hospitalisation) among women undergoing birth at home. The y-axis shows the percent of the total costs for all women included in the study, and the x-axis shows the percent of the women in home birth

20% of the women who gave birth at home accounted for 41% of the total time costs for the midwives (Fig. 2). Furthermore, we see that the 10% with the lowest time costs accounted for only 3.2% of the total costs. In comparison, the 10% with the highest costs accounted for 25% of the total costs. This finding means that the 10% with the highest costs are 7.8 times as costly as the 10% with the lowest costs. We did not include transport costs and hospital costs for any admissions in these calculations.

### Uncertainty and sensitivity analysis

#### Uncertainty estimated using a probabilistic sensitivity analysis

The figures in Additional file 5 illustrate the uncertainty for the results by showing how likely different results are according to the simulations made. A probabilistic sensitivity analysis (PSA) based on parameter uncertainty was used. According to the credibility intervals (CrIs) in Table 6 for all the comparisons between alternatives, we can conclude that the differences in costs of home births when compared with hospital births that appeared by chance was less than 2.5%.

#### Deterministic sensitivity analysis

In the deterministic sensitivity analysis, we assessed the effect of changes in assumptions or the level of input data to the model for the cooperation of the low-risk hospital birth alternative (A1) and the planned home birth alternative (B1) as shown in Table 8.

**Table 8:** Sensitivity analysis for changes in the planned home birth costs

Change analysed	Change in B1 costs (€)
i) Visit by paediatrician at home after home birth lasts 2 h instead of 1 h	86.8
ii) The rate of caesarean sections for planned home birth (B1) increase to the level for low-risk hospital birth (A1)	101.2
iii) Midwife no. 2 involved in all births and not just 52.5% of home births (B1)	168.1
iv) Adds an extra hour for both midwives in scenario iii above	91.3
v) The midwives’ birth-bag and equipment are used in 20 births, not in 40	7.2

To estimate the effects of increasing the rate of caesarean sections among women who planned for a home birth, we increased the rate to be equal to the rate for low-risk births in a hospital (change from 0.01867 to 0.0587). For the average woman planning to give birth at home, the costs increased by 101.20 Euro. Some of the reason for this modest effect is that only 8.71% of the women in alternative B1 were hospitalised, and those who changed to caesarean sections “come from” vaginal birth, which also is a relatively expensive treatment.

The changes shown in Table 8 have effects that will probably not change the conclusions that we can draw based on Table 6. Even if we accept the changes, we tested the sensitivity for the likelihood that the costs of a home birth alternatives B1, B2, and B4 were less than the costs of hospital birth alternatives A1 and A2, which was less than 2.5% according to the CrIs. This situation is still the case if we combine changes iii and iv (168.1€ + 91.3€ = 259.4€) as shown in Table 6.

A difference between the groups giving birth at home versus in hospital in terms of education, age, and whether the woman has given birth before was found. These results are presented in Additional fil 2, Table S1. Because our data consist of several data sets, we could not make an overall correction for this situation in the simulation model. Nevertheless, we could estimate what effect these differences would have on midwives’ time use by calculating how the time used for home birth would change if the women who gave birth at home was the same as the women who gave birth in a hospital. We performed this calculation using the analysis presented in Additional fil 2, Table S1, which shows how age, education, and parity affect the total hours spent by the midwife. We found that only education and parity had a significant effect on the total hours. In Additional fil 2, Figure S1, we see how these factors affect the midwives’ use of time, and we used this effect as a proxy for the importance of these factors. If the education level among those who give birth at home was in line with that of those who give birth in hospital, the hours spent by the midwives would have been reduced by 0.813 h per birth. The corresponding correction for the proportion who had previously given birth caused an increase in the midwives’ time use by 1.265 h, which together with the correction for education, yielded a total effect of 0.452 h. This value is 2.1% of the midwives’ average hours spent per home birth, which was 21.5 h (excluding the midwives’ travel time). Furthermore, we estimated that the costs associated with the midwives’ time use were approximately 0.63 of the total costs of a planned home birth (B1). If we corrected for the two variables in the group who gave birth at home, the costs would have increase by approximately 1.3% (2.1% x 0.67). We can see from Tables 6 and 8 that this increase in cost alone did not change the conclusion that alternatives B1, B2, and B4 have lower costs than both A1 and A2.

Furthermore, for the results in Table 6, we assumed that no woman or baby spend less than 24 hours in hospital after giving birth. We performed a sensitivity analysis for this assumption, and the results of this analysis do not appear to have any bearing on the conclusions we drew (Additional file 6).

### Discussion

We found that the costs of planned home births were 45.9% of low-risk hospital births. If we assumed a model in which designated midwives are responsible for facilitating home births and these midwives are on call for one birth at a time (B3), we see that the costs per home birth would be 35.7% higher than giving birth in hospital. If a midwife group of a certain size cooperates and is responsible for all home births in a region and these midwives are on call for several women at the same time (B4), the home-birth costs represent 69.7% of the costs of giving birth in a hospital.

### Strengths and limitations of the study

A strength of the study is our access to three different comprehensive datasets on resource use, one of which has been conducted specifically for this study (dataset i in Fig. 1). The datasets have not only contributed to good estimates of expected values but also to calculations of their statistical distributions. The use of multiple datasets was possible through our use of a model approach, which also provided good opportunities to incorporate many different data sources for unit costs.

To enhance transparency, we practised detailed reporting of the in-data to the simulation model. Further, we had a comprehensive strategy for handling uncertainty by using deterministic (one-by-one variable and scenarios) and probabilistic sensitivity analysis. An overview of the distributions used in the PSA can also be found in Additional file 3.

Another strength is that the women who were included in the home birth groups were to a large extent women actually giving birth at home because the classification was based on which women entered into contracts with their midwives as late as in week 37 (alternative B1). Nevertheless, 8.2% of the women were transferred to hospital before the birth and gave birth at the hospital. We therefore conducted separate analyses for the group of only women who actually gave birth at home (alternative B2).

Even though home births in Norway are not organised by the health authorities, a scheme in which the government pays midwives to assist with a home birth is in place. These payments are normally between 730 and 1,500 Euros. The woman herself must pay the midwives for being on call and for their transport costs. This payment normally ranges from €216 to €1,294 (NOK 3,000 to NOK 18,000). A strength in our analysis was not focusing on transfer of money between different participants (or actors) but rather estimated the socio-economic costs of home birth compared to giving birth in a hospital.

A final strength is that we analysed the consequences of organisational solutions for home birth, which seems to have a major impact on the profitability of home birth. For example, the effects of possible on call costs were analysed. These costs have not been analysed explicitly in any other study.

By using a CMA, we limited ourselves to comparing the costs of different alternatives. Based on extensive international literature in the field, we assumed that the health effects/outcomes for mother and child are the same whether the relevant groups give birth at home or in hospital. Our choice of research design are thus in line with several comparable health economic evaluations in this field [5, 7, 8, 19, 20].

It is difficult to conduct a randomised controlled trial for home versus hospital birth for both practical and ethical reasons. For our observational data, comparability between the groups giving birth in hospitals versus at home can therefore be questioned. In addition, the data were obtained from different time periods. To explore this possible problem, we performed sensitivity analyses in which we adjusted for statistical differences between the groups, and we used conservative estimates of complications for hospitalised women who planned to give birth at home. We assumed that all these hospitalised women experience complications that lead to higher hospital costs than the minimum costs of hospital births. Furthermore, we performed analyses in which entire scenarios/alternatives for hospital births were assumed to be without caesarean sections and complications (alternative A2). However, none of these alternative or sensitivity analyses changed the main conclusions of the study.

The midwives included in the study of resource use in home births (data set i in Fig. 1) were not randomly selected but rather selected based on a list of midwives and only included midwives that had completed at least 10 home births. However, Table 1. shows that the women they assisted (data i) had the same characteristics as the women planning for home birth in Blix et al. [9] (data ii) in terms of age, education, and number of previous births.

The microsimulation model used to estimate on call costs for alternatives B2 and B3 was based on our expert opinion about what is the most common compensation midwives receive per hour on call in Norway (see Table 7). The uncertainty this contributes to is in addition to the parameter uncertainty that appears in the PSA. Therefore, in Table 7, we show the effects of different estimates for compensation.

### Discussion of the results

The costs of an *actual home birth* (alternative B2) was 35.1% of a vaginal birth without complications in hospital (alternative A2). This finding coincides well with Anderson and Anderson (5), who found these costs to be 32.8% for the United States. A similar result was obtained by Hendrix et al. (7) from the Netherlands concerning the actual place of birth (Table 8 in their article), and Cicero et al. (8) from Italy found 60.9% and 49.7%, respectively.

We estimated the costs of *planned home birth* (alternative B1) to be 45.9% compared to low-risk birth in hospital (alternative A1), while Janssen et al. (20) found this percentage to be 47.2% in Canada for 0–28 days during/after birth. Schroeder et al. [21] found 68.0% in England (Table 2 in their article) for the period of intrapartum and immediate after birth. Other articles, such as those by Hendrix et al. [7] (Table 5 in their article) and Hitzert et al. [6] (Table 3 in their article) are less comparable. In these articles, the women were divided into groups based on the women’s early preferences for birth at home or at a hospital, and thus, a large proportion of those in the home birth group ended up giving birth in hospital, and the home birth group was charged for this hospital costs in the analysis (see the Introduction). Hu et al. [22] use a population-based microsimulation for Queensland, Australia, and find that a home birth costs 37.1% and 83.5% compared to current standard care from a public hospital perspective and a public hospital funders perspective, respectively.

Our findings discussed above do not *include costs associated with on call* and therefore would be appropriate for healthcare systems in which home births are organised by large hospitals. If we assume instead that midwives facilitating home births work alone or in pairs, which has been common practice in Norway, on call costs should also be included. In such cases (alternative B3), we found that a home birth costs 35.7% more than a low-risk birth in a hospital (A1). As far as we know, only the article by Cicero et al. [8] explicitly mentions on call costs as part of the analyses. They analysed out-of-hospital deliveries in a private regime in which it was assumed that for every birth, two midwives were on call from week 37. They found that a home birth would then cost 58.8% more than a hospital birth, which was defined as spontaneous birth without complicating diagnosis. In our analyses, we assumed that an average of 1.52 midwives were present during the birth and thus 1.52 also on call before the birth. If we instead assume that two midwives were on call in our analysis per birth (the costs increases by 3.0%) and compared this cost with our alternative vaginal birth without complications in the hospital (A2 in Table 6), the corresponding percentage for Norway would be 46.4%. As we can see, both Cicero et al. [8] and our results show that if on call costs were included under these assumptions, the cost picture would change markedly.

As mentioned, some uncertainty associated with the compensation we assumed to be consistent with the welfare loss midwives experience by being on call is present. If, for example, compensation was set too high in our analyses, the costs of home birth would have been estimated correspondingly too high. The level at which alternative B3 goes from costing more to costing less than a low-risk hospital birth is when the on call costs overall for midwives was € 2204 (30,665 NOK). So, if the midwives overall find that a lower sum than this in terms of welfare will compensate them fully for being on call for a birth, B3 can be considered cost-effective compared to low-risk hospital birth (A1).

We assumed that on call costs consist only of the midwives’ welfare loss. We then assumed that being on call would not lead to a change in production for the midwives in, for example, the health service (apart from the time they spend helping the woman in labour, which is accounted for). If this assumption does not hold, these costs (lost production) would be added to the costs we already estimated.

On call can be organised in different ways, such as organising a group of midwives who share the responsibility of being on call for several women at a time. If we compare a low-risk hospital birth with a planned home birth in this way and according to the Swedish model from the Karolinska Hospital (alternative B4), the costs of a home birth would then be 69.7% that of a low-risk birth in hospital. We have not found articles whose results are relevant to compare with these findings.

When generalising our results to other countries, certain factors should be considered. In our data, home births were in the context of a continuity-of-care model in which the women were assisted by a known midwife. Midwifery-led continuity-of-care models are associated with less interventions and complications compared with other models [23].

In our analyses, we have considered that the rate of caesarean section is somewhat lower among those who planned a home birth (alternative B1: caesarean section rate was 1.87%) than those who planned for hospital birth (alternative A1: caesarean section rate was 5.87%). The caesarean section rate in Norway has been stable between 15% and 17% over the last two decades [11]. Thus, these differences in caesarean section rate have less effect on our estimates than for countries in which the caesarean section rate generally is higher. It could then be assumed other countries’ rates of caesarean sections that are higher than those in Norway would contribute to home births when compared with hospital births and would be somewhat less costly than we have found.

Over time, certain changes take place, and many of the figures we have used are from before 2018. For example, we found that an average of 1.52 midwives were present during labour. In the future, it may be a requirement that always two midwives are present during the actual birth at home. For alternative B1, we found that such a change would increase costs by 9.0%.

It is often difficult to transfer the costs from one country to another due to different living costs. We therefore used PPP to convert from the Norwegian kroner (NOK) to the Euro. In addition, we estimated the percentage of costs of one treatment performed by another. Thus, we neutralised the problems of different cost levels to a certain degree.

To better generalise the results to other countries, we calculated the effect for different assumptions and for different ways of organising maternity care for home births. If these processes were organised as part of the offer from the large hospitals, alternatives B1 and B2 would probably be the most relevant. If these midwives were organised individually, in pairs, or in smaller groups, B3 and B4 would probably be the most relevant alternatives.

### Impact on policy and practice

The results from our study are useful for health authorities and others organising home births as we provide estimates of the costs associated with home birth services.

When operating the midwife service for home births in a private regime, it seems especially important to pay attention to the costs associated with being on call, which seem to have a strong economy of scale.

### Further research

The costs related to being on call were analysed to a very small extent, while under certain regimes, such costs can be a decisive cost component. More research on both how on call costs should be analysed, and how these costs affects the cost-effectiveness of home births should be conducted.

## Conclusions

Home birth is cost-effective if the midwives facilitating home birth are organised into larger groups, or they work as part of hospitals that also facilitate home births.

Midwives working separately or in pairs to assist with home births and are on call for one birth at a time, will normally not be cost-effective if the costs of being on call are included. Despite this lack of cost-effectiveness, home birth in Norway has been cost-effective from the perspective of the health service budget because most of the on call costs have been covered by the midwives themselves.

How the health services for home births are organised largely determines whether home births will be cost-effective compared to hospital births.

## Supplementary Information

Supplementary Material 1.

Supplementary Material 2.

Supplementary Material 3.

Supplementary Material 4.

Supplementary Material 5.

Supplementary Material 6.

## Data Availability

Requests for data can be sent to the authors. Software used: For modelling the model we used Excel (Office version 2306), and @Risk 7.6 for Excel from Palisade was used for performing the PSAs.

## Abbreviations

A1: Low-risk birth in hospital
A2: Vaginal birth in hospital
B1: Planned home birth
B2: Home birth
B3: Planned home birth + on call for single women
B4: Planned home birth + on call for groups
BMI: Body Mass Index
CI: Confidence interval
CM: Contract meeting
CMA: Cost-minimisation analysis
CrI: Credibility interval
DRG: Diagnostic-related group
DSA: Deterministic sensitivity analyses
€: Euro
GLM: Generalized linear models
GP: General practitioner
Km: Kilometer
no/cc: No complications or comorbidities
NOK: Norwegian kroner
OLS: Ordinary least squares
OUS: Oslo University Hospital
PPP: Purchasing power parity
PSA: Probabilistic sensitivity analyses
SE: Standard error
w/cc: With complications or comorbidities
