# Supplementary material for: Cost minimisation analyses of birth care in low-risk women in Norway: a comparison between planned home birth and birth in a standard obstetric unit
Source: BMC Health Serv Res. 2024 Sep 30;24:1150. doi: 10.1186/s12913-024-11631-7 (PMC11440651; doi:10.1186/s12913-024-11631-7)
Supplement: Supplementary file 1 — Supplementary Material 1. [file 12913_2024_11631_MOESM1_ESM.pdf]

Additional file 1: Two questionnaires used for collecting data on time used and costs of home birth.

HOME BIRTHS AND THE USE OF RELATED RESOURCES<br>Questionnaire to midwives attending homebirths.<br>(Translated from Norwegian).
Information about the study<br><br>We appreciate your participation in this evaluation concerning resources related to home births. This survey is part of an evaluation of activities and costs related to home births. The data will be used to compare costs for home births to costs at a low-risk obstetric unit in hospital. The results will be presented as a scientific publication.<br><br>We hope you will answer the attached questions for your latest 10/20 women where you, as midwife, were assisting in planned home births, one questionnaire for each woman. Denote each woman with an unique number starting with 1.<br><br>You can access the survey by clicking the link at the end of this e-mail. You do not have to complete all the questionnaires in one operation, but record as many as you want and continue the rest when suited.<br><br>Participation in this study is voluntary and you can withdraw your consent at any given point prior to closing the database. You do not have to explain/justify the reason for withdrawal.<br><br>Due to the quality and representativity of the study, it is important that you fill in the electronic questionnaire for the relevant women. It is estimated that we will collect data on 300 women that have given birth at home, according to the number of midwives we have addressed in this study.<br><br>We recognize that some of the questions could be difficult to answer in a precise manner, and we ask you to make estimations when needed.<br><br>All information will be treated confidentially and will only be used in statistical analyzes. Your answers will be anonymized (links to names will be deleted and any indirect personally identifiable information will be categorized to secure anonymity) shortly after we receive them and no later than November 30th 2017. *No* names should be recorded on the questionnaire for the relevant women.<br><br>This study is approved by the national data protection officer at the Norwegian Agency for Shared Services in Education and Research (SIKT, former NSD).<br><br>Any questions regarding this trial can be addressed to Anette S. Huitfeldt (mobile: 922 34 667 or e-mail: anette.s.huitfeldt@gmail.com) or to Pål Joranger (mobile: 922 93 776 or e-mail: paal.joranger@hioa.no).<br><br>Good luck!

Questions:	Answer options:
Write your initials	
Repeat your initials	
Denote each woman by numbers, not by letters. If this is your first woman, you write 1, if it is the second, you write 2 and so on.	Numbers from 1 to 60
The woman's age at delivery	< 20 years<br>21-24<br>25-29<br>30-34<br>35-39<br>>40
What was the woman's highest completed education?	Primary school<br>High school<br>University/college 4 years<br>University/college >4 years
Was this woman nulli- or multiparous?	Nulliparous<br>Multiparous
Did you make a house call for this woman in week 36/37 (possibly another time) to sign a labour contract?	Yes<br>No<br>Do not remember
If you made a house call, how many midwives attended?	1 midwife<br>2 or more midwives<br>Other
If you wrote «other» in the question above, how many attended?<br>(It may be a group of midwives on call making separate house calls)	
How long time did you spend on this house call prior to home birth?	0-15 minutes<br>16-30<br>31-45<br>46-60<br>1 hour-1hour 15 min<br>1 hour 15 min-1 hour 30 min<br>1 hour 30 min-1hour 45 min<br>1 hour 45 min-2 hours<br>2 hours-2 hours 15 min<br>2 hours 15 min-2 hours 30 min<br>2 hours 30 min-2 hours 45 min<br>2 hours 45 min-3 hours<br>More than 3 hours
Did you accompany this woman to the hospital for an outpatient assessment prior to home birth?<br>For example, for a post term assessment.	Yes<br>No<br>Do not remember
If yes on the question above:<br>How long time did you spend on this assessment including the journey to and from the hospital and any waiting time at the hospital?	

Record the estimated hours used on this assessment.	
How long time did you use for transportation from your place of residence to the woman's home?	0-15 minutes<br>16-30<br>31-45anette<br>46-60<br>1 hour-1hour 15 min<br>1 hour 15 min-1 hour 30 min<br>1 hour 30 min-1hour 45 min<br>1 hour 45 min-2 hours<br>2 hours-2 hours 15 min<br>2 hours 15 min-2 hours 30 min<br>2 hours 30 min-2 hours 45 min<br>2 hours 45 min-3 hours<br>More than 3 hours
How many kilometers was it from your place of residence to the woman's home? How many kilometers was it from your place of residence to the woman's home?	
Did you make a "blind trip" to this woman?<br>"False alarm", for example if the woman was in early labour or her contractions decreased.	Yes<br>no
If you answered «yes» on the question above, how many times did you visit the woman prior to home birth?	1 time<br>2 times<br>3 times<br>4 times<br>Other
How long time did you spend on this "blind trip"? Sum up the time spend for all "blind trips" in whole hours.	
How many midwives attended the home birth?	1 midwife<br>2 or more midwives
Midwife number 1 during birth:<br>How long did you stay with the woman, from your arrival until you left after birth?<br>Record the time spent in whole and half hours, 5,5 denotes five and a half hours, 12,0 denotes twelve hours	
Midwife number 2 during birth:<br>How long did you stay with the woman, from your arrival until you left after birth?<br>Record the time spent in whole and half hours, 5,5 denotes five and a half hours, 12,0 denotes twelve hours	
Was the woman transferred to the hospital?	Yes<br>No

If the woman was transferred to the hospital, how many kilometres were there from the woman's residence to the hospital?<br>Record the number of kilometres in whole numbers.<br>How many house calls did you make after birth for this woman, and how long time did you spend on these house calls?<br>This concerns both women who gave birth at home and those who were transferred to the hospital but got house call from the home-midwife after birth.	
Home visit number 1	0-15 minutes<br>16-30<br>31-45<br>46-60<br>1 hour-1hour 15 min<br>1 hour 15 min-1 hour 30 min<br>1 hour 30 min-1hour 45 min<br>1 hour 45 min-2 hours<br>2 hours-2 hours 15 min<br>2 hours 15 min-2 hours 30 min<br>2 hours 30 min-2 hours 45 min<br>2 hours 45 min-3 hours<br>More than 3 hours
Home visit number 2	-------"--------------
Home visit number 3	-------"--------------
Home visit number 4	-------"--------------
Home visit number 5	-------"--------------
Home visit number 6	-------"--------------
Where was the baby examined by the pediatrician?	At home<br>In hospital<br>The baby was not examined by a pediatrician
Did the women use a birthing pool and in case, how did she get hold of it?	She did not use a birthing pool.<br>She hired a birthing pool.<br>She bought a birthing pool.<br>She borrowed one for free or used a pool she already had.
Did you have other expenses than presented in this survey, please describe.	
Thank you for taking time answering this survey.	
