# Supplementary material for: Cost minimisation analyses of birth care in low-risk women in Norway: a comparison between planned home birth and birth in a standard obstetric unit
Source: BMC Health Serv Res. 2024 Sep 30;24:1150. doi: 10.1186/s12913-024-11631-7 (PMC11440651; doi:10.1186/s12913-024-11631-7)
Supplement: Supplementary file 2 — Supplementary Material 2. [file 12913_2024_11631_MOESM2_ESM.pdf]

Additional file 2: The importance of women's age, education, and parity

We performed subgroup analysis based on alternative B1 to see how the composition of the groups of women giving birth could affect the use of resources. The analyses of the midwives' use of time on the women who gave birth at home were based on generalized linear models (GLM) with gamma distribution family and log-link function. This choice is based on Deb et al. (1) and Glick et.al. (2). For comparison with the GLM analyses for the midwives' time use, ordinary least squares (OLS) analyses are also performed, and these largely coincide in terms of p-values.

For some of this analysis, we also used the "Two-part model", where the first step is based on "probit" and the second step on GLM with gamma "distribution family", log-link function and "robust" estimation of variance. The choice is based on recommendations in Deb et al. (1). In step 1, the analyses tell us what effects an event to occur; for example, when midwife number 2 attends and contributes during childbirth. For our example, step 2 tells us about what affects how long midwife no. 2 stays, given that she has attended. All the analyses were based on "Exclude cases listwise" in case of missing data. The analyses were performed in SPSS 28 and Stata 17.

We see from Table S1 that midwives spend significantly more time with women with higher education and on first-time mothers. This is exclusive travel time. In Figure S1, we see the estimated marginal values for time use where the midwives spend 26.6 hours (95% CI 22.8–30.4) in total per woman if they have a high education and are first-time mothers and spend 17.9 hours (95% CI 15.6–20.1) if the woman has a low education and has given birth before. That is a difference of 8.7 hours (44%).

Table S1. Time spent by the midwives and women's age, education, and parity.

Variable	Total time use, Midwife		Time in contract meeting (CM)		Time for meetings between CM and birth		Time on "false alarms"		Time for birth, no. 1 and 2 midwives		Time for meeting after birth	
	Coef.	Sig.	Coef.	Sig.	Coef.	Sig.	Coef.	Sig.	Coef.	Sig.	Coef.	Sig.
Age	-.016	.837	.038	.592	-.175	.416	-.110	.667	.027	.705	-.007	.950
Education	.171	.025	.130	.087	.564	.024	-.215	.417	.115	.139	.186	.087
Previous birth	-.228	.005	.072	.364	.006	.980	-.529	.034	-.418	.000	-.066	.576
Constant	3.121	.000	.743	.000	.359	.145	.860	.007	2.516	.000	1.173	.000
N	354		354		354		354		354		354	

We used «generalised linear models» (GLM) with gamma «distribution family» and log-link function.

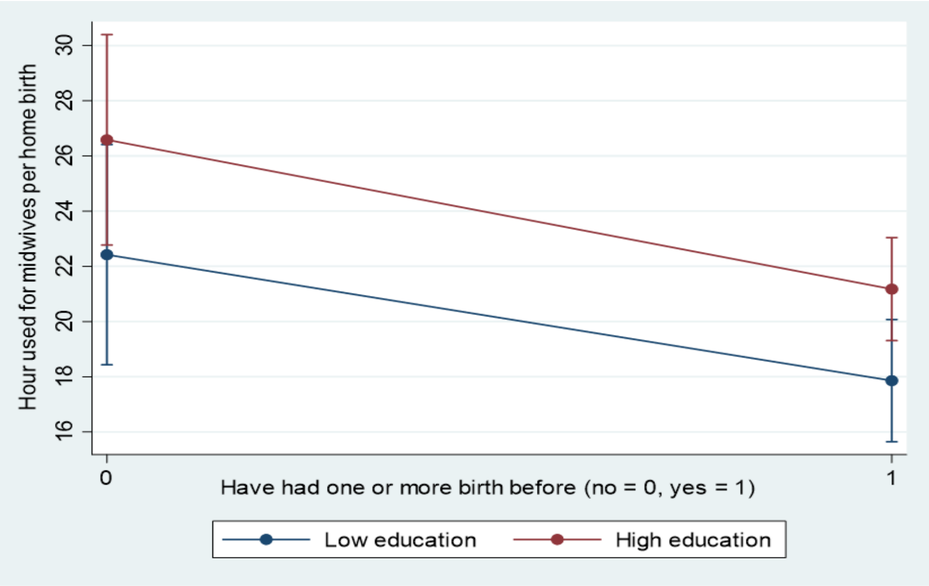

Figure S1: Estimated marginal values for time used by different values for education and whether one has given birth before or not (exclusive travel time and hospitalisation). The point estimates' 95% confidence interval is shown by vertical lines.

Furthermore, we see that the time spent at a contract meeting is not significantly affected by the three covariates (Table S1). Time spent in meetings after this and until the birth, on the other hand, is significantly affected by the level of education. Whether one has given birth before significantly affects the time spent on the blind trips carried out.

From Figure S2, we see that the estimated marginal values for midwives' time use for the birth is 9.1 hours (95% CI 8.4–9.7) if the woman has given birth before, and 13.8 hours (95% CI 12.1–15.4) if not. This is a difference of 4.7 hours (52% increase).

However, what determined the participation of midwife no. 2 at birth? In our analysis with the "Two-stage model", none of the three covariates were significant (p = 0.96) for whether midwife no. 2 participated or not (stage 1 for the two-part model). On the other hand, among those who participate, how long midwife no. 2 contributes (stage 2 for the two-part model) is significantly (p <0.0005) longer for first-time mothers than for those who have given birth before.

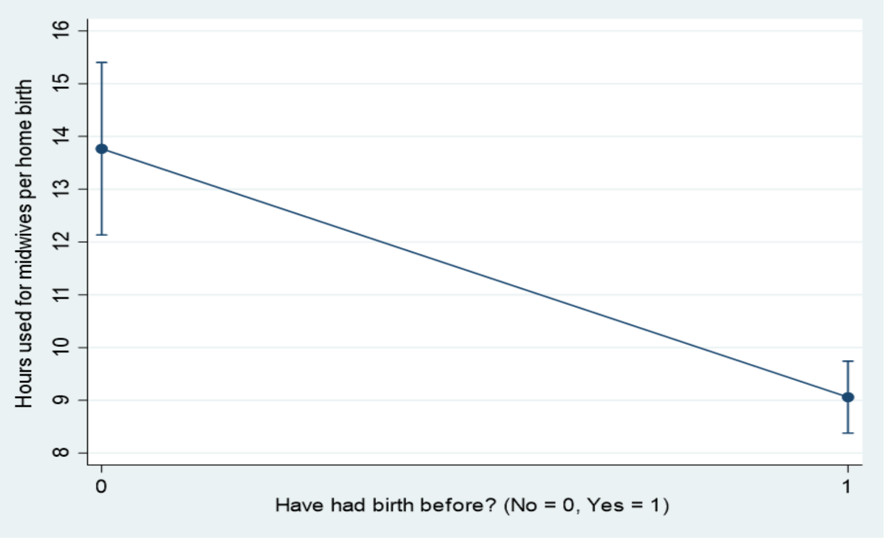

Figure S2: Estimated total time used for midwives 1 and 2 on the birth, related to whether the woman has given birth before or not (exclusive travel time and hospitalisation). The point estimates' 95% confidence interval is shown by vertical lines.

References

1. Deb C, E.C.Norton, Manning WG. Health econometrics using Stata. Texas: Stata Press; 2017.
2. Glick HA, Doshi JA, Sonnad SS, Polsky D. Economic Evaluation in Clinical Trials. Second ed. Oxford: Oxfors University Press; 2015. 252 p.
