# Supplementary material for: Cost minimisation analyses of birth care in low-risk women in Norway: a comparison between planned home birth and birth in a standard obstetric unit
Source: BMC Health Serv Res. 2024 Sep 30;24:1150. doi: 10.1186/s12913-024-11631-7 (PMC11440651; doi:10.1186/s12913-024-11631-7)
Supplement: Supplementary file 3 — Supplementary Material 3. [file 12913_2024_11631_MOESM3_ESM.pdf]

Additional file 3: The distribution type and parameters used in the PSA

All the estimation of uncertainty was performed by combining Excel (Office version 2306) with @Risk 7.6.

Table S1: Distribution types and parameters of resources used for planned home birth (the base case).

Parameter	Type of distribution, probability	Distribution parameters, probability (alfa; beta)	Type of distribution, time used per meeting/ activity	Distribution parameters, time used per meeting/ activity (alpha; beta)
**Birth at home**				
Time used for transport to the woman			Gamma	466.46; 0.0737
Km from the woman in labour to the Hospital			Gamma	31.770; 0.6699
Check by a paediatrician at home	Beta General	81.88; 221.948		
Contract meeting (CM), probability	Beta General	341.383; 10.958	Gamma	966.285; 0.0027
Meetings between CM and birth				
1st meeting	Beta General	149.854; 203.575	Gamma	897.541; 0.0020
2nd meeting	Beta General	103.783; 249.221	Gamma	478.792; 0.0035
3rd meeting	Beta General	79.004; 270.591	Gamma	343.962; 0.0046
4th meeting	Beta General	44.440; 305.156	Gamma	233.697; 0.0073
5th meeting	Beta General	15.384; 326.487	Gamma	69.662; 0.030
False alarm	Beta General	113.576; 236.041	Gamma	147.174; 0.0267
The birth:				
Midwife 1		No parameter uncertainty	Gamma	1294.640; 0.0055
Midwife 2	Beta General	144.45; 130.693	Gamma	595.484; 0.0097
Meetings after birth:				
1st meeting	Beta General	339.861; 9.88019	Gamma	1824.97; 0.0009
2nd meeting	Beta General	233.086; 116.543	Gamma	1337.004; 0.0012
3rd meeting	Beta General	104.562; 244.641	Gamma	511.422; 0.0037
4th meeting	Beta General	29.609; 319.796	Gamma	411.432; 0.0075
5th meeting	Beta General	10.856; 338.556	Gamma	76.085; 0.0387
6th meeting	Beta General	1.975; 347.617	Gamma	76.085; 0.0387
Other time used by the midwife per birth	Beta General	199.692; 146.692	Gamma	245.041; 0.0112

Table S2: Distribution types and parameters used for resource used data for planned hospital birth and for transference and hospitalisation of women and children with planned home birth.

Parameter	Type of distribution	Distribution probability parameters (alfa; beta)
**Transfer to hospital for planned home birth**		
Urgent hospitalisation before birth, mother	Beta General	41.646; 436.278
Children hospitalised	Beta General	10.967; 470.033
For those transported to hospital:		
Use taxi	Beta General	0.693; 44.307
Use ambulance	Beta General	15.921; 29.079
Use ambulance helicopter	Beta General	1.386; 43.614
Hospital, for the transferred mothers before birth:		
Caesarean section no/ccb (DRG 371)	Beta General	6.941; 25.447
Vaginal delivery w/cc (DRG 372) (unconditional probability: 0.0127)	Beta General	5.007; 29.285
Hospital, for the transferred child:		
Newborn, birth weight 1500–2499 g or other immaturity, without multiple prob. (DRG 388B) (unc. prob.:0.00220)	Beta General	0.98; 48.02
Urgent hospitalisation of mother after birth	Beta General	11.977; 469.023
**Planed for birth at hospital**		
Hospitalised mother:		
Caesarean section no/cc (DRG 371)	Beta General	90.926; 1458.07
Vaginal delivery w/cc (DRG 372)	Beta General	60.827; 1484.78
Hospitalized child:		
Newborn, birth weight 1500–2499 g or other immaturity, without multiple prob. (DRG 388B)	Beta General	5.996; 1543.00

All simulation was done in NOK (before recalculated to Euro), and the parameter in Table 4.3 is then related to NOK.

Table S3: Distribution type and parameters used for unit costs used in the cost analysis.

Parameter	Unit or DRG score used	Type of distribution	Distribution parameters (alfa; beta)
Midwife time cost	Cost/hour	Gamma	384.16; 1.326
GP	Cost/hour	Gamma	384.16; 2.207
Transport to the woman, car cost	Cost/visit (both way)	Gamma	254.02; 0.511
Transport to hospital when emergency:			
Ambulance	Cost/transport	Gamma	95.99; 76.656
Helicopter	Cost/transport	Gamma	96.03; 600.864
**Hospital costs, mother**:			
Caesarean section no/cc	1.416 DRGs	Gamma	96.04; 0.0147
Vaginal delivery w/cc	0.95 DRGs	Gamma	96.04; 0.0099
Vaginal delivery no/cc	0.628 DRGs	Gamma	96.04; 0.0065
**Hospital costs, child:**			
Healthy newborn, no/cc	0.574 DRGs	Gamma	96.04; 0.0060
Newborn, birth weight 1500-2499g or other immaturity, without multiple prob.	2.933 DRGs	Gamma	96.04; 0.0305
**Medicine and equipment:**			
Surgical drapes, number used	Number	Gamma	8.644; 0.3471
Sterile gloves, number used	Number	Gamma	15.366; 0.1302
Anti-RH IMMUNOGLOBULINS, probability for use in a home birth	Probability	Beta general	3.357; 30.2170
Birth set, unit price	Cost/birth set	Gamma	15.366; 260.308
Birth set, number of births in use	Number	Gamma	15.366; 2.603
