# Supplementary material for: Cost minimisation analyses of birth care in low-risk women in Norway: a comparison between planned home birth and birth in a standard obstetric unit
Source: BMC Health Serv Res. 2024 Sep 30;24:1150. doi: 10.1186/s12913-024-11631-7 (PMC11440651; doi:10.1186/s12913-024-11631-7)
Supplement: Supplementary file 4 — Supplementary Material 4. [file 12913_2024_11631_MOESM4_ESM.pdf]

Additional file 4: Costs of medicine and equipment for home birth

Table S1 The resource use and unit costs for medicine and equipment.

	Unit price, €	Probability for use in a home birth	Amount/number used if this is used in a birth	Price per birth, €
**Assistance material**				
Not sterile latex gloves	17.90	1	0.04	0.72
Sterile gloves, price per pair	1.00	0.8	2	1.60
Sterile gauze, cotton wool, compress	6.40	0.9	1	5.76
Surgical drapes 100x100 (10 pcs)	8.62	1	3	25.86
Disposable catheters (10 pieces)	56.06	0.05	0.1	0.28
Acupuncture needles, price per pack of 100 needles	3.67	0.2	0.05	0.04
Sewing threads (10 pcs. in the package)	17.90	0.3	0.1	0,54
Syringe tip for vitamin K, price per tip	2.80	0.98	0.01	0.03
Syringe tip for oxytocin, price per 100 tips = 39	2.80	0,.25	0.01	0.01
Syringe tip for vitamin K to all children and oxytocin, price for 1 ml syringe 100 pieces	7.04	1.23	0.02	0.17
Ultrasound Gel, Greenscan, one tube	6.83	0.9	0.020	0.12
Hibitane 1% vaginal cream 250ml	13.66	0.9	0.020	0.25
**Medicines**				
Physiological saline, 1000 ml x 10	18,46	0.02	1.00	0.37
Oxytocin, 5 IE, 10 stk. × 1 ml	27,85	0.25	0.1	0.70
Anti-RH IMMUNOGLOBULINS, 625 IE/ml, 2 ml, includes syringe tips	45.74	0.1	1	4.57
Vitamin K, 10 mg/ml, 5 × 0.2 ml, glass ampoule	10.12	0.98	1	9.92
**Birth set**	Total price	Number of births in use		
Sewing items, “Lærdals bag”, children's scales, etc.	287.52	40		7.2
**Total cost**				**58.10**
