# Supplementary material for: Cost minimisation analyses of birth care in low-risk women in Norway: a comparison between planned home birth and birth in a standard obstetric unit
Source: BMC Health Serv Res. 2024 Sep 30;24:1150. doi: 10.1186/s12913-024-11631-7 (PMC11440651; doi:10.1186/s12913-024-11631-7)
Supplement: Supplementary file 5 — Supplementary Material 5. [file 12913_2024_11631_MOESM5_ESM.pdf]

Additional file 5: Distributions for the results from the PSA

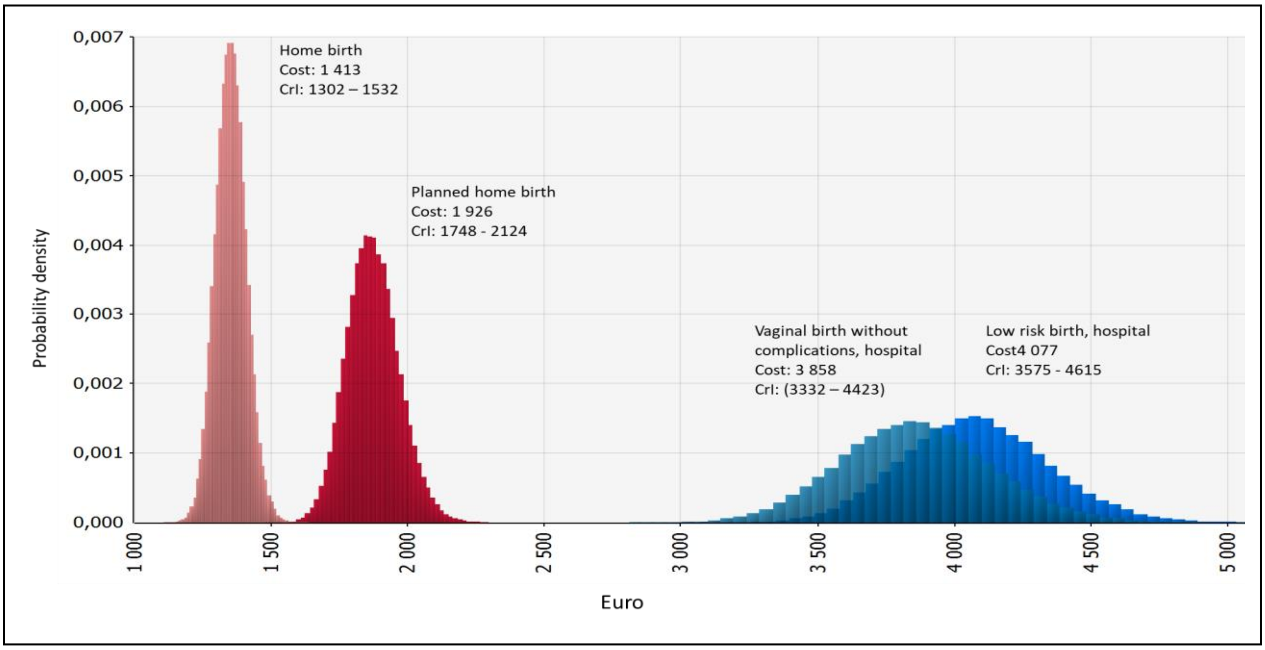

Figure S1. The distribution of the results from the PSA for the two alternative births in hospital (A1 and A2) and for the two home birth alternatives without on-call cost (B1 and B2). CrI is the credibility interval, which shows the 2.5-th and 97.5-th percentile of the outcome distribution.

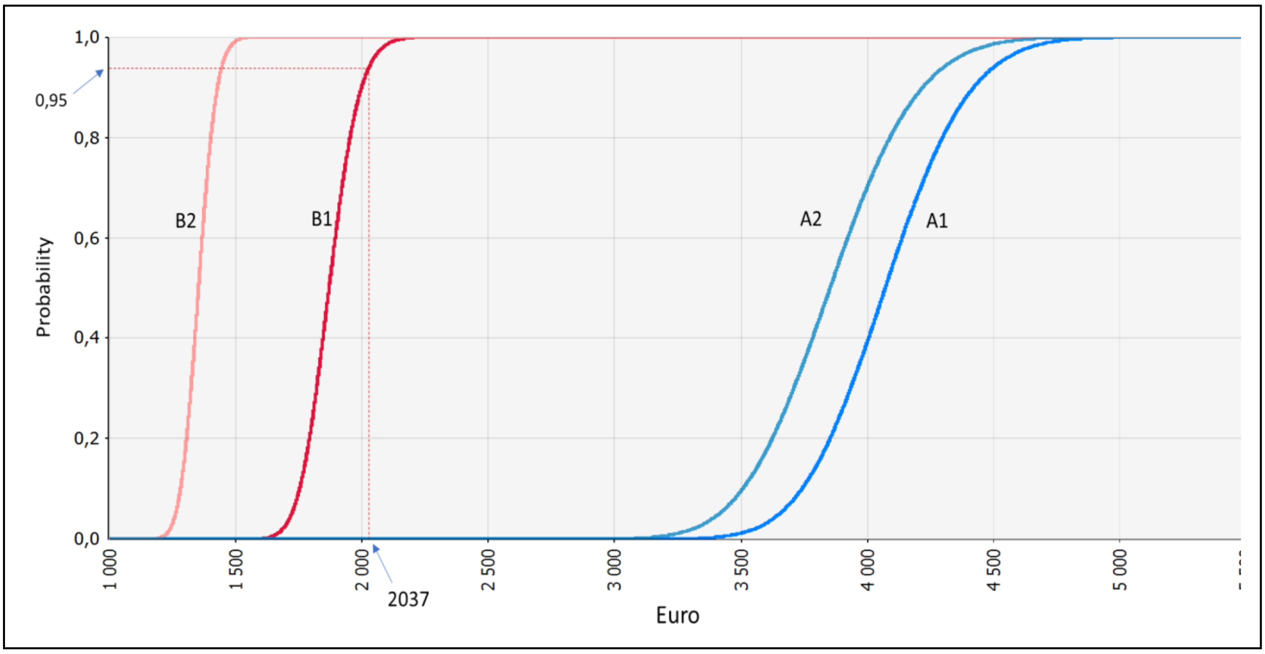

Figure S2. The cumulative probability and costs for the two alternatives for birth in hospital (A1 and A2) and the home birth alternative including on-call cost (B1 and B2).

Figure S2 shows the probability that the costs of an alternative are less than a given cost level per birth. For example, there is a probability of 0.95 that the cost of alternative B1 is less than €2037 per birth. That means it is only 5% likely that costs are greater than €2,037 per birth.

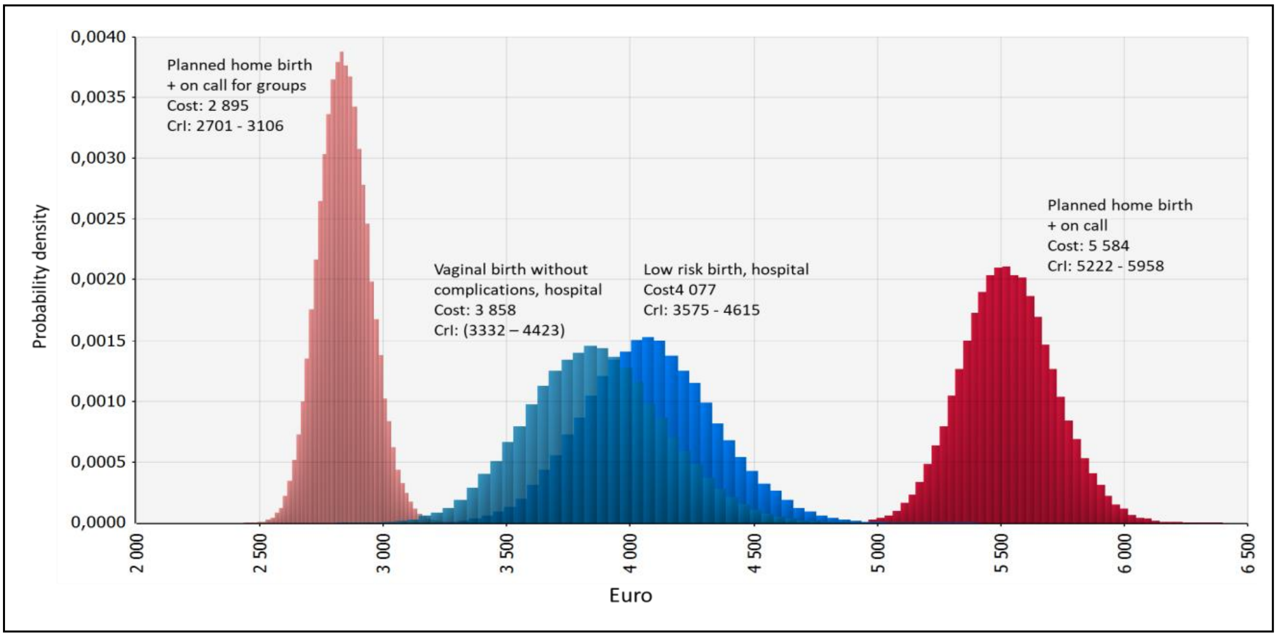

Figure S3. The distribution for the results from the PSA for the alternative birth in hospital (A1 and A2) and for the home birth alternative with on-call cost included (B3 and B4).

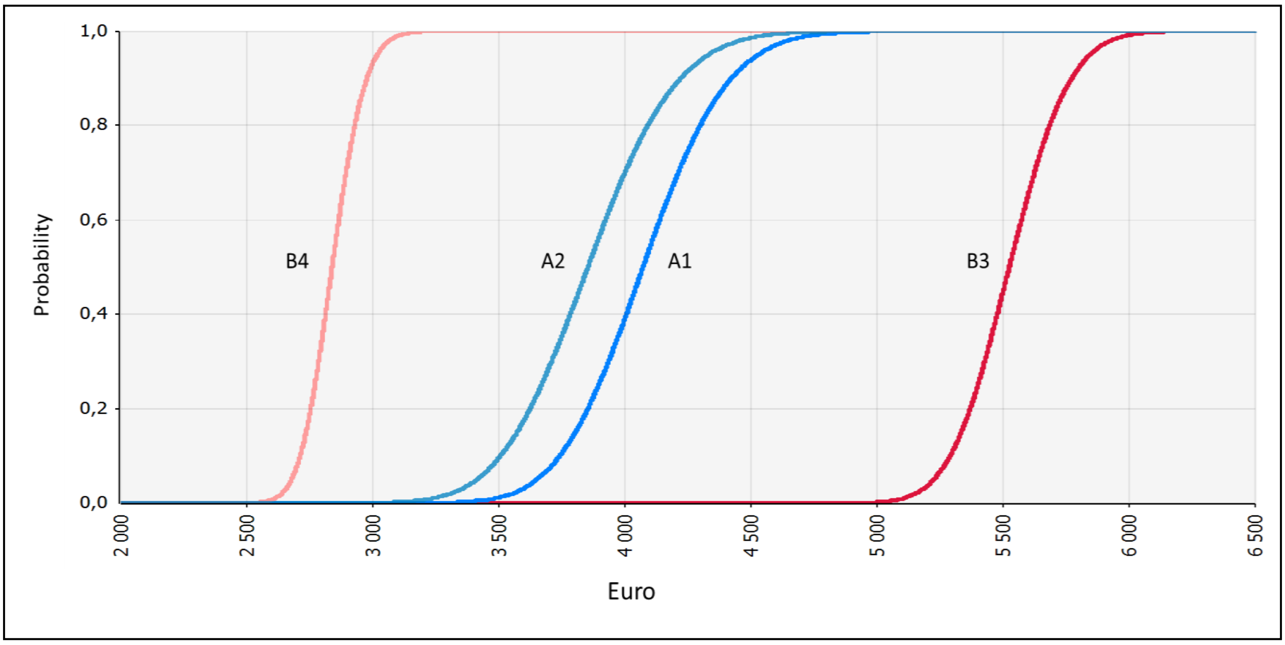

Figure S4. The cumulative probability and costs for the two alternatives for birth in hospital (A1 and A2) the home birth alternative, including on-call cost (B3 and B4).
