# Supplementary material for: Cost minimisation analyses of birth care in low-risk women in Norway: a comparison between planned home birth and birth in a standard obstetric unit
Source: BMC Health Serv Res. 2024 Sep 30;24:1150. doi: 10.1186/s12913-024-11631-7 (PMC11440651; doi:10.1186/s12913-024-11631-7)
Supplement: Supplementary file 6 — Supplementary Material 6. [file 12913_2024_11631_MOESM6_ESM.pdf]

## Additional fil 6: Sensitivity analysis regarding the assumption of being hospitalized less than 24 hours.

For the analyses yielding the results in Table 6, we assumed that no woman or baby spent less than 24 h in the hospital after birth. We made this assumption because it is very unusual to be discharged before 24 h, and we lack data for this in our three data sets (i, ii and iii). We conducted a sensitivity analysis of how large deviations in the results these simplifications may contribute. We received data about the length of stay from a Norwegian hospital. We chose to use the years that were unaffected by the Covid pandemic and therefore used the data for 2018–2020.

We found that among women who underwent vaginal birth, an average of 1.12% were admitted less than 24 h after childbirth (if we use the data for 2018–2022, this percentage would be 1.03%). If we correct for the 5.78% of the women who had planned to give birth in hospital but had a caesarean section, we find that for all women who had planned a hospital birth, 1.06% stayed less than 24 h after birth.

If we also consider the proportion of women hospitalised for less than 24 h, the costs of low-risk birth in hospital (A1) were reduced by 33.51 Euros. This means a 0.82 percent reduction in the costs of «Low-risk birth in hospital» (A1). These reduced costs have a marginal impact on the comparisons and the conclusions made in the article.

There is some uncertainty associated with this sensitivity analysis. First, we have no data on any increase in costs because of extra mother and/or child readmissions. Furthermore, we assumed that all women with planned home births who nevertheless give birth in hospital are hospitalised for more than 24 h. These factors indicate that the sensitivity analyses performed in this study show larger deviations than we expected. In addition, there is some uncertainty regarding the representativeness of the data used.
